# Molecular diversity analysis, drought related marker-traits association mapping and discovery of excellent alleles for 100-day old plants by EST-SSRs in cassava germplasms (*Manihot esculenta* Cranz)

**DOI:** 10.1371/journal.pone.0177456

**Published:** 2017-05-11

**Authors:** Bin Wang, Xin Guo, Pingjuan Zhao, Mengbin Ruan, Xiaoling Yu, Liangping Zou, Yiling Yang, Xiao Li, Deli Deng, Jixiang Xiao, Yiwei Xiao, Chunji Hu, Xue Wang, Xiaolin Wang, Wenquan Wang, Ming Peng

**Affiliations:** 1 College of plant science & technology, Huazhong Agricultrural University, Wuhan, Hubei, PR China; 2 Institute of Tropical Bioscience and Biotechnology, Chinese Academy of Tropical Agricultural Sciences, Haikou, PR China; 3 Key Laboratory of Biology and Genetic Resources of Tropical Crops, Ministry of Agriculture, Haikou, PR China; National Bureau of Plant Genetic Resources, INDIA

## Abstract

Cassava is the third largest food crop of the world and has strong ability of drought tolerance. In order to evaluate the molecular diversity and to discover novel alleles for drought tolerance in cassava germplasms, we examined a total of 107 abiotic stress related expressed sequence tags—simple sequence repeat (EST-SSR) markers in 134 cassava genotypes coming from planting regions worldwide and performed drought related marker-traits association mapping. As results, we successfully amplified 98 of 107 markers in 97 polymorphic loci and 279 alleles, with 2.87 alleles per locus, gene diversity of 0.48 and polymorphic information content (PIC) of 0.41 on average. The genetic coefficient between every two lines was 0.37 on average, ranging from 0.21 to 0.82. According to our population structure analysis, these samples could be divided into three sub-populations showing obvious gene flow between them. We also performed water stress experiments using 100-day old cassava plants in two years and calculated the drought tolerance coefficients (DTCs) and used them as phenotypes for marker-trait association mapping. We found that 53 markers were significantly associated with these drought-related traits, with a contribution rate for trait variation of 8.60% on average, ranging between 2.66 and 28.09%. Twenty-four of these 53 associated genes showed differential transcription or protein levels which were confirmed by qRT-PCR under drought stress when compared to the control conditions in cassava. Twelve of twenty-four genes were the same differential expression patterns in omics data and results of qRT-PCR. Out of 33 marker-traits combinations on 24 loci, 34 were positive and 53 negative alleles according to their phenotypic effects and we also obtained the typical materials which carried these elite alleles. We also found 23 positive average allele effects while 10 loci were negative according to their allele effects (AAEs). Our results on molecular diversity, locus association and differential expression under drought can prove beneficial to select excellent materials through marker assisted selection and for functional genes research in the future.

## Introduction

Cassava, an important food and starch crop, has excellent adaptability to multiple environments. However, seasonal drought every year reduces its yield in major planting regions [1, 2]. One approach to solve this problem is to develop new cultivars which are not only drought-tolerant but also have high yield.

Analysis of the genetic diversity of germplasms is a valuable tool for cassava breeding, especially when aiming for drought tolerance [2]. High genetic diversity in germplasm improves the probability to select excellent new germplasms. Genetic diversity is also used as a reference to select materials with outstanding characteristics, such as high yield and drought tolerance, and parent materials with excellent breeding potential and multiple elite alleles of interest.

DNA markers have become a powerful tool for genetic diversity analysis of germplasms and for discovering alleles of interest. Single nucleotide polymorphism (SNP) chips, such as GoldenGate Infinium from Illumina, SNPStream from Beckman Coulter or GeneChip from MegAllel and Affymetrix [3], and high throughout sequencing methods, such as reduced-representation sequencing, restrict site associated DNA (RAD) or exon trapping sequencing, are the two major techniques used in modern molecular marker technology. While GBS strategy become more and more popular for its cheaper and massive information, SSR markers remained to be widely used in MAS and primary mapping researches. At present, SSR markers that detect co-domainance and higher polymorphisms are also valid alternative. EST-SSRs are designed on expressed sequences which involved in the variety of metabolic functions [4]. Gene-based SSR markers are located in target genes, returning clear information the same as EST-SSRs. Thus, both of EST-SSRs and gene-based SSRs had potential use in quantitative trait loci (QTL) mapping and association mapping researches.

Molecular marker technology has also rapidly developed in cassava, with more and more studies using EST-SSRs in combination with other molecular markers such as genomic SSRs [5], amplified fragment length polymorphism (AFLP)[6] or EST-SNPs [7]. This allowed constructing cassava linkage maps in F1 generation segregation population derived from heterozygote parents. Gene-based SSRs have also been used in cassava researches. A previous report studied 846 putative SSRs in 8577 cassava unigene sets and evaluated 124 new, unique polymorphic gene-based SSRs in 25 cassava cultivars and their wild relatives [8]. Moreover, SNP genotyping methods were also used in cassava [9, 10]. For all this, SNP chips in cassava genetic linkage and physical mapping, genotype-by-sequencing (GBS) approaches in re-sequencing cassava cultivars and their wild ancestor, together with the announcement of a database containing cassava genome and SNPs information database derived from 53 cassava cultivars promoted molecular marker development in cassava [11–13].

Linkage QTL mapping and association mapping are used to explore novel alleles in crops such as rice, maize, wheat, cotton, soybean, rape and sugar beet. However, their application in cassava is still rare. The major method to explore novel alleles in cassava is linkage QTL mapping in F1 generation derived from heterozygous parents. Previous studies used this for yield related traits [6, 14], starch pasting viscosity [15], cyanogenic glucoside [14, 16] and cassava mosaic disease (CMD) resistance related traits [17, 18]. Due to the high frequency natural cross-pollination in cassava, the presence of just a few flowers and a high rate of heterozygosis, it is difficult to cultivate homozygote lines and it is impossible to fine map them using high generation backcross populations as done in other crops. Even so, application of association mapping methods in natural population to discover novel alleles of important agronomic traits in cassava might be a viable option.

Because of the complexity of drought stress responses and the bottle necks of breeding methods based on phenotype selection, researchers need to consider QTL mapping in drought tolerance breeding projects [19]. In order to analyze population structure, association mapping of drought tolerance related traits and excellent alleles for drought tolerance in available cassava germplasms, we genotyped 134 germplasms by using 107 EST-SSR markers. Moreover, we used reverse genetics data to confirm our association mapping results. Finally, we obtained a series of casual associated loci with elite alleles and their carriers.

## Materials and methods

### Plant materials

The 134 cassava accessions used in our study were obtained from 11 countries and areas as described in S1 Table. They were major cultivars or breeding materials in these countries or areas which could be considered as hybrids and were vegetative reproduction by stems. All of these accessions were used as the basic population to analyze genetic diversity, water depress experiment and marker-traits association mapping. Two cassava cultivars Arg7 and SC124 which had different growing strategies under drought stress [20] were used to perform water depress experiment for qRT-PCR verification.

### Water stress experiments, phenotyping and statistical methods

In order to evaluate the drought tolerance of cassava germplasms, we preformed water stress experiments in the year 2014 and 2015 in Haikou, Hainan province, China. Stems of cassava germplasms which were harvested in January in 2014 were cut into equal length fragments and grown in flowerpots which were filled with 1:2 ratio of sand to soil. Water depress experiment was performed after 100 days accessions were planted. Plants with even growth potential were chosen for water stress experiments. Drought treatment group and control group contained six plants respectively and be divided to two repeats. The former was not watered for 12 days while the latter continued to be watered. Three plants of both two repeats with uniform phenotypes were taken as samples from drought treatment group and control group respectively. Above-ground fresh weight (AGFW), storage root fresh weight (SRFW), storage root number (SRN), storage root dry weight (SRDW) and storage root dry matter percentage (SRDMP) per plant were measured. And leaves and root were cut into pieces and mixed to put into the liquid nitrogen and then turn to ultra-low temperature freezer (-80°C). Samples were used to test the physiological traits containing content of proline, soluble reducing sugar (SRS) and malondialdehyde (MDA) per unit fresh weight and activity of superoxide dismutase (SOD), peroxidase (POD) and catalase (CAT) per unit fresh weight. The measure method of proline content is ninhydrin colorimetry, anthrone colorimetry for SRS content, thiobarbituric acid chromatomety (TBA) for MDA content, nitroblue tetrazolium reduced (NBT) method for SOD activity, guaiacol method for POD activity and hydrogen peroxide (H_2_O_2_) ultraviolet (UV) absorption method for CAT activity. And all of tests for 6 physiological phenotypes were performed by using respective assay kit (Comin biotechnology Co., ltd. Suzhou, China). We used only 95 germplasms in 2015 to water depress experiment due to limited breeding stems and low seedling emergence rate of some germplasms. And seven traits of these cassava genotypes, including AGFW, Proline, MDA, SRS, SOD, POD and CAT, were also detected in 2015. All phenotypes were turned into drought tolerance coefficients (DTC), as described before [21]. And then, maximum, minimum, average, standard deviation and coefficient variation were analyzed by SPSS18.0 software (http://www-01.ibm.com/software/analytics/spss/). Boxplot figures of DTCs were drawn by using GraphPad Prism version 5.0 for Windows, GraphPad Software (San Diego, California, USA, www.graphpad.com)

### DNA extraction and SSR genotyping

Young leaves of 134 accessions were used for genomic DNA as described before [5]. We chose 107 pairs of SSR markers, containing 55 pairs of EST-SSRs [5] and 52 pairs of gene-SSRs [8], to genotyping cassava germplasm using a previously described PCR program [22]. PCR products were mixed with a florescent dye to perform capillary electrophoresis. SSR fragments were assigned to genotypes according to the method reported by Wang et al. [23].

### Genetic diversity analysis

Number of alleles per locus, major allele frequency, gene diversity and polymorphic information content (PIC) were calculated using Powermarker Version 3.25 software [24]. Genetic coefficient between every two genotypes was calculated using NTSYSPC2.10e software [25]. Analysis of molecular variation and sub-population genetic differences (PhiPT) and gene flow (Nm) was performed using Genalex 6.2 [26].

### Population structure analysis

In order to analyze the population genetic structure and deal with its interference for association mapping, STRUCTURE V2.0 software [27, 28] was applied to analyze the genotype data of cassava germplasms. Both of burnin and MCMC were set to 100,000 and K ranged from 1 to 10 with three integrations for every K value. LnP(D) and Variance of LnP(D) which were calculated by STRUCUTRE software were used to estimate the ΔK for every K value as previously described [29]. Two curve graphs were drawn that LnP(D) and ΔK ranged with K value increasing from 1 to 10 respectively. The real K value was that one co-responding the first tunnel of ΔK. These three Q matrices of the real K values, estimated by using STRUCTURE, were combined with CLUMPP 2.0 [30]. The combined Q matrix was used as covariance matrix in association mapping and source data to draw the stacked column chart of Q matrix in excel. Besides, all of these 134 genotypes were divided into three clusters and mixed cluster according to the method reported by Yang et al. [31].

### Marker-trait association mapping

DTCs of traits obtained in 2014 and 2015 were used as the phenotypes in association mapping while the SSR fragments information of germplasms as genotypes. Marker-trait association mapping was performed in TASSEL V2.1 [32] by using Q + K+ MLM with MAF > 0.05 filter condition. We obtained significance *P* values and phenotypes variation R^2^ and *P* values were modified according to the FDR method [33]. Marker-trait associations were considered significant if the FDR of the *P* values were lower than 0.05. All of significant associated markers were mapped in the *Manihot esculenta* Cranz genome v6.1 (https://phytozome.jgi.doe.gov/pz/portal.html)[13] and were used to draw a graph of distributions of the associated markers by MAPChart V2.0 software [34] according to the physical position of 107 EST-SSR markers.

### Expression analysis of associated genes

In order to confirm whether associated genes were actual functional genes, gene codes in the cassava genome v6.1 and v4.1 were used to search the transcriptomics under water stress, obtained from genebank and containing the controls and drought data of three genotypes: SC124, Arg7 and W14, sequenced by Illumina HiSeq 2000 by the Institute of tropical bioscience and biotechnology, Chinese Academy of Tropical Agricultural Sciences, Haikou, PR China (Accession numbers of genebank, SRR2360410, SRR2361999, SRR2388947, SRR2388956, SRR2404199, SRR2404206, SRR2495946, SRR2495947, SRR2495949, SRR2495950, SRR2496326, SRR2496093) [35] and the iTRAQ-based proteomics database [20]. Log2 based FPKM change fold values equal to FPKM of associated genes under drought condition based on the transcriptomics reported by Wei et al. [35] were calculated divide them for the controls and used to draw a heat map using the MeV2.0 software [36].

Furthermore, in order to confirm significantly different expressed genes, relative expression level of the 24 genes which were selected from previous omics database were detected in two cassava cultivars Arg7 and SC124 by qRT-PCR method. Samples which were used to qRT-PCR experiment derived from independent drought stress experiment of cassava cultivars. Every pot contained both of the two cultivars plants to keep them in coincident water depression. The conditions of plants growing, methods of drought treatments and tissues collection were the same as described before. Tissues of every cultivar were collected from the mixed materials of five independent plants in both of the drought treatment groups and control groups respectively. Total RNA extraction and cDNA first strand reverse experiments were performed by using RNAprep Pure Plant Kit (Polysaccharides&Plolyphenolics-rich, Tiangen Biotech, BeiJing Co. Ltd) and FastQuant RT Kit (with gDnase, Tiangen Biotech, BeiJing Co. Ltd) repectively. All of primers used to amplify target genes were shown in S2 Table. House-keeping gene *MeActin* with JGI ID code Manes.12G150500 was used as internal control and three technique repeats were performed for every sample. qRT-PCR performed by using SYBR Premix Ex TaqTM II (perfect real time) kit (Takara Biotechnology, Dalian, China) on StepOne plus system. RQ was estimated by StepOne software v2.1 through 2^-ΔΔCT^ method based on C_T_ values [37]. Significance level of relative expression between drought treatment groups and control groups were calculated by two-tailed *T* test method in Microsoft excel. And differential expression genes were selected with P<0.05 at least in one tissue vs. cultivars.

### Discovery of elite alleles

Our association mapping and expression analysis indicated that there were total twenty-four genes significantly associated with drought tolerance. Phenotypic effects of the alleles at these loci were estimated as previously reported [38] and alleles whose phenotypic effects were positive would be considered positive alleles, while alleles whose phenotypic effects were negative values would be considered as negative alleles. The average positive (negative) allele effect of the locus (AAE) was also calculated according to a previously reported method [38]. Loci of which AAE had positive values were considered positive loci of associated traits; otherwise they were counted as negative loci.

## Results

### Genetic diversity analysis

To evaluate the molecular diversity and genetic structure of cassava germplasm resources, we used 107 EST-SSR markers, 98 of which were located in cassava chromosomes (S1 Fig.) while others were mapped in scaffolds of cassava genome version 6.1. Ninety-eight genes were aligned and other nine markers were not successfully mapped. Finally, Ninety-eight SSRs primers couples successfully amplified 97 polymorphic loci and 279 alleles. The MAF per locus was 0.62 on average, ranging from 0.29 to 0.99. The number of alleles per locus was 2.87 on average, ranging from 2 to 8. Gene diversity per locus was 0.48 on average, ranging from 0.02–0.80. Average PIC value per locus was 0.41, ranging from 0.02 to 0.77. Genetic coefficient between every two accessions was 0.37 on average, with a maximum of 0.82 measured between Xinxuan048 and Royang9-1 and a minimum of 0.21 between ECU81 and R7. Genetic coefficients between every two lines were widely ranged, indicating that the genetic relationships between germplasms were very complex.

All of 134 germplasms were divided into four groups according to their area of origin (Asia, South America, Central America and Africa). As was shown in Table 1, result of AMOVA analysis estimated that molecular variations were present mainly within subpopulations. However, the PhiPT was 4.00E-3 with a P value of 0.41), indicating that this was not significant. The gene flow (Nm) was 116.87. These data could indicate that there was no significant subpopulation differentiation among the 134 cassava germplasms and that the gene flow was very strong among subpopulations.

**Table 1:** AMOVA of cassava germplasm resources.

Source	df	SS	MS	Est. Var.	%
Among Pops	3	87.741	29.247	0.115	0.4
Within Pops	126	3377.212	26.803	26.803	99.6
Total	129	3464.954		26.918	100%

### Population structure

**Fig 1:**
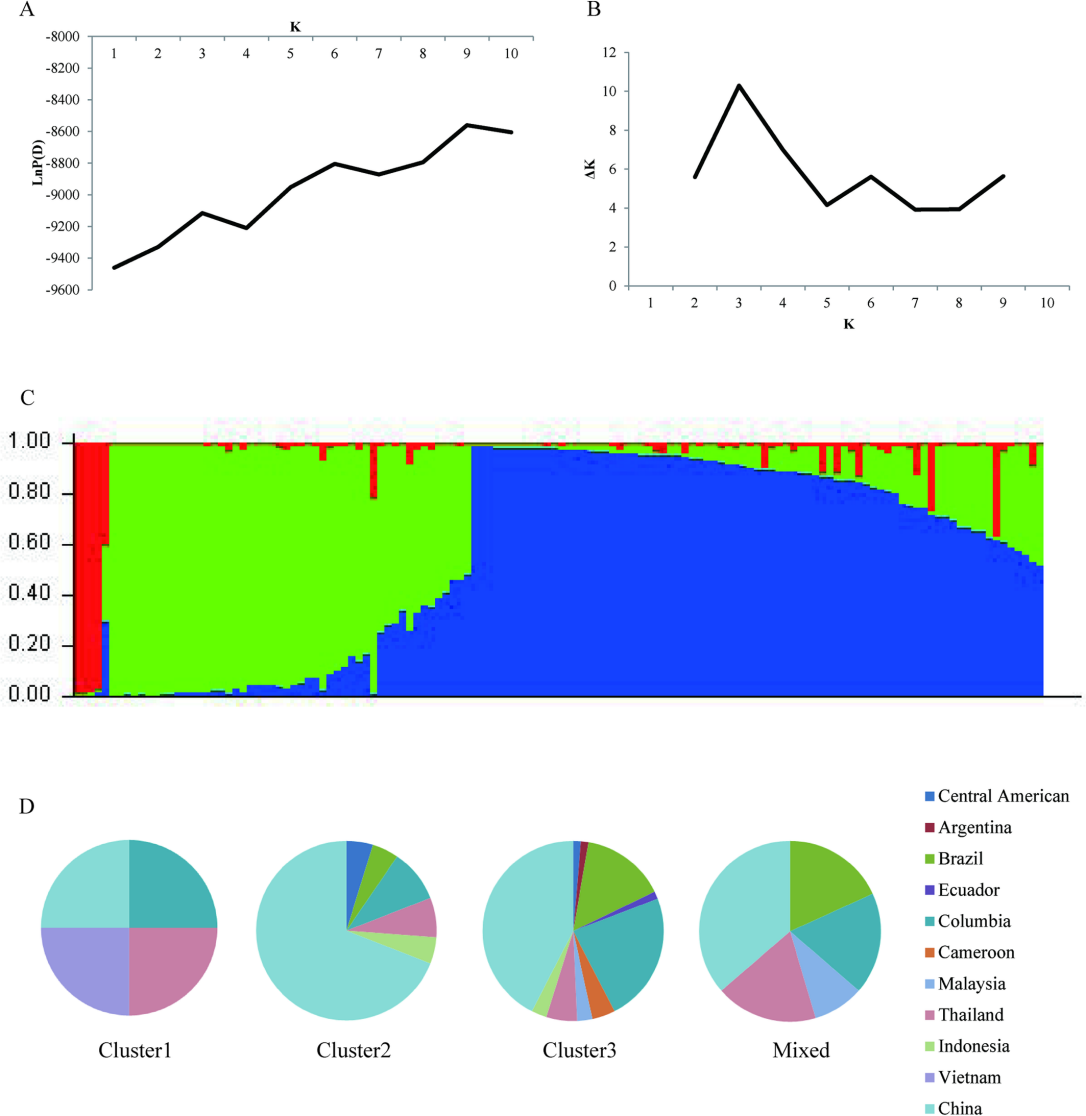
Population structure analysis. A, LnP(D) value graphed against K from 1 to 10. B, ΔK values at K from 1 to 10. C, stacked column chart of Q matrix. D, Origin distributions of the three clusters and mixed cluster.

In order to distinct the genetic structure of the 134 germplasms, we used the Structure software to estimate the number of sub-populations. As shown in Fig 1A and 1B, LnP(D) was the first significant peak at K = 3 while the ΔK was also the first tunnel and peak value at K = 3. Thus, total 134 germplasms were divided into three clusters. Then, we obtained the results of three integrations of K = 3 and combined the Q values of every germplasms into one Q matrix using CLUMPP V2.0 (Fig 1C). We divided the sub-groups using the STRUCTURE software according to previously reported (Yang et al. 2011), obtaining a number of genotypes for the three clusters and mixed cluster of 4, 46, 74 and 10, accounting for 3.00%, 34.33%, 55.22% and 7.46% of the total, respectively. AMOVA for these three clusters, performed by using the Genalex software, showed that PhiPT was 0.03 (*P* = 0.02) and Nm was 18.73, indicating that these three clusters were significantly different with a strong gene flow. However, we found no significant association between the three clusters and their original countries or areas (Fig 1D).

### Summary of description statistics for drought-related traits

**Fig 2:**
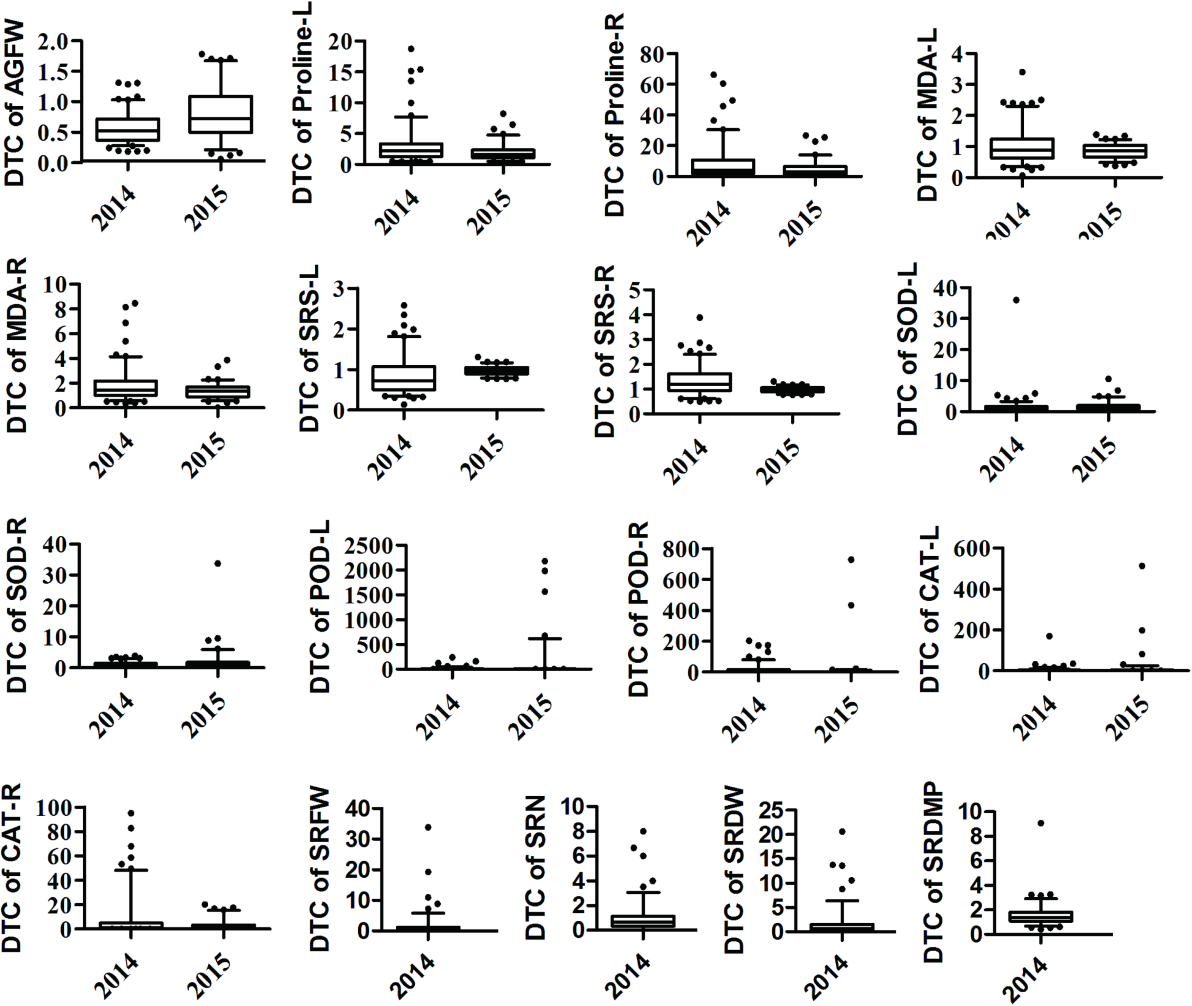
Boxplots of DTCs of cassava germplasm phenotypes in water stress experiments.

In order to evaluate the drought tolerance of cassava germplasms, we preformed water stress experiments in the years 2014 and 2015, as described in the methods section. We tested seven physiological phenotypes of 100-day old plants, including AGFW, Proline, SRS and MDA content, as well as SOD, POD and CAT activity. Storage root-related traits of 100-day old plants were only tested in 2014 (Fig 2). Using the DTC definition reported by [21], we translated these phenotypes into the respective DTCs and analyzed the results using the descriptive statistics function of the SPSS software (S3 Table). All of these traits were qualitative traits with a continuous distribution character. The maximum DTC range was that of POD in leaves in 2015 while the minimum was that of SRS in leaves in 2015. Coefficient DTC variation were very different for different traits. Among them, the biggest CV % value was that of DTC in leaves in 2014 and the smallest CV % value was that of SRS in leaves in 2015. This indicated that DTCs were very distinct in different germplasms.

### Marker-traits association mapping

**Fig 3:**
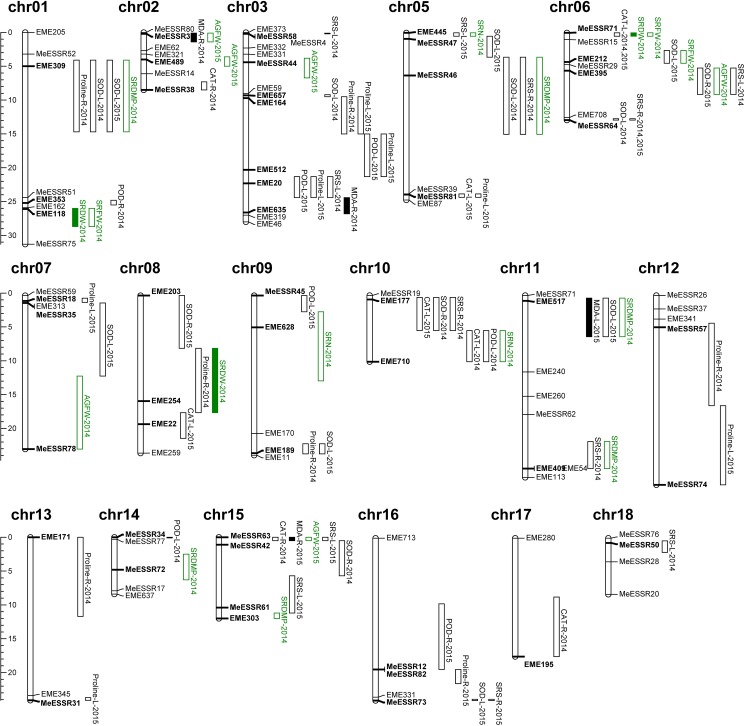
Distribution of associated markers in cassava genome (P<0.05).

To explore novel allele for drought tolerance in cassava, we performed marker-traits association mapping by using the Q+K+MLM model. As shown in S4 Table, fifty-three pairs of EST-SSR markers, located in 17 chromosomes of cassava except the 4^th^ chromosome (Fig 3), were associated to DTCs of drought tolerance related traits (*P_FDR*<0.05). Explanation of phenotypic variation (R^2^) was 8.60% on average, ranging from 2.66% to 28.09% (S4 Table). Twenty-two marker-trait combinations had an explanation of phenotypic variation of them higher that 10% and accounted for the 22% of total significant marker-trait combinations. In our two-year water stress experiment, 3 SSRs, EME309, MESSR64 and MESSR71, were associated with the same traits and organs. Another marker, EME164, was associated with the same traits but in different organs. In 2014, we detected 47 marker-trait combinations of 33 loci, of which ten associated to multiple phenotypes while 23 others were associated to a single phenotype. In 2015, forty marker-trait combinations of 31 loci were associated, with eight of them that were multiple-association while 23 were associated to one phenotype. Besides, there were 13 markers that were associated not only to the physiological traits but also to biomass and storage root related traits of 100-day old plant.

### Expression pattern analysis

**Fig 4:**
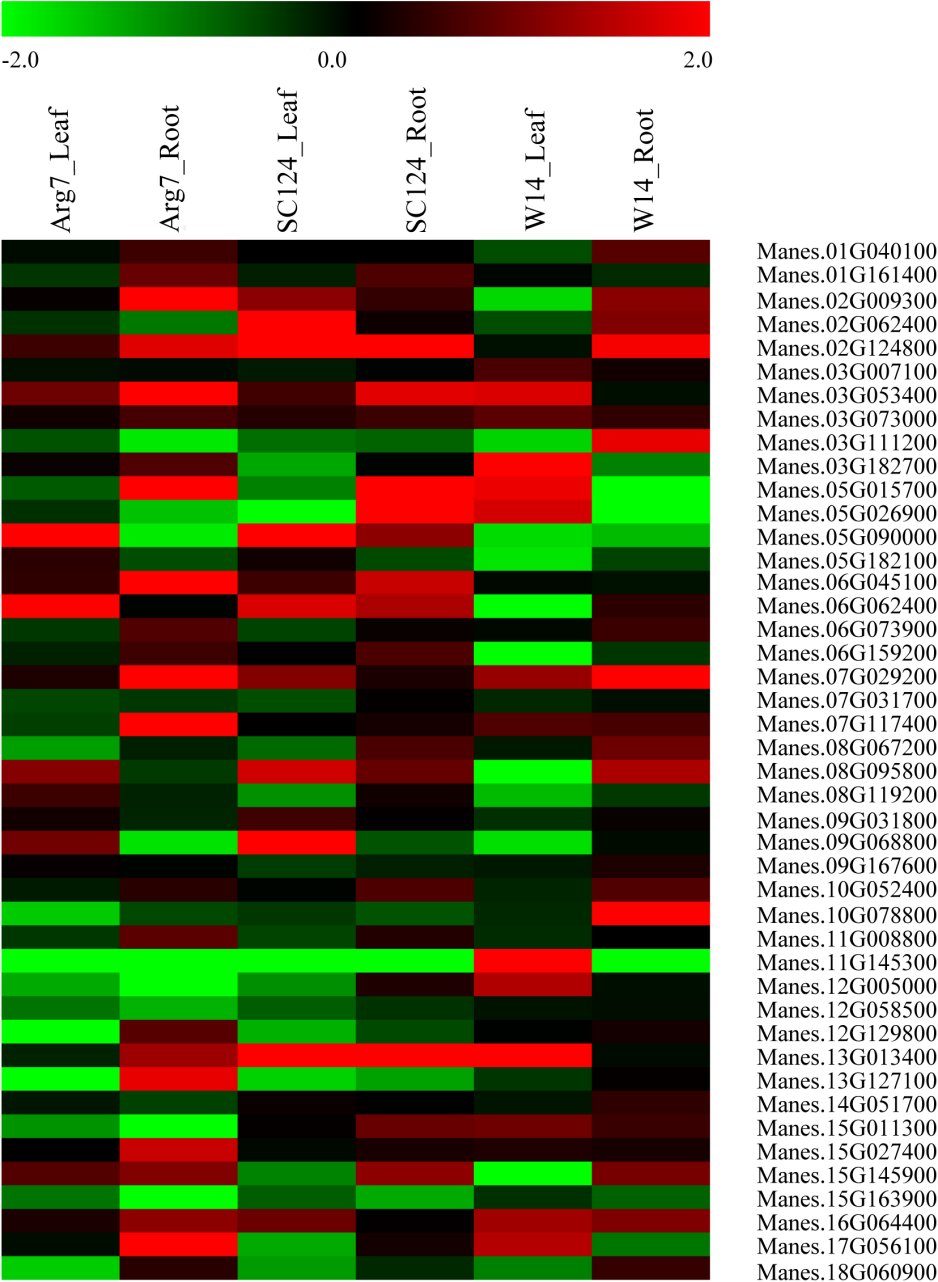
Expression profiles of associated genes in cassava. Log2 based FPKM change fold values drought stress plants by respective control value were used to create the heat map. The differential expression thresholds of significant up and down regulation were set to 2.0 and -2.0 respectively.

In order to confirm these genes co-localized with associated EST-SSRs and to discover the real functional genes, these 53 associated makers were mapped in the cassava genome. 51 of them were located in coding genes in cassava genome version 4.1 and 6.1 (S4 Table). 44 genes were also present in cassava RNA sequencing data in control and water-stressed cassava plants derived from genebank. We used Log2 based on FPKM change fold values under water stress to draw the heat map, with 2.0 and -2.0 as the upper and lower limit respectively. As results, 24 genes that were differently expressed at least in one sample vs. cultivar under drought stress and control condition (Fig 4). And according to the protein database reported by previous study [20], four proteins encoded by four associated genes differential expressed in response to water stress in at least one organ, cultivar or time point (Table 2). According to the transcriptomics and proteomics database of cassava water stress plants, there were four genes that were associated to water stress as for DNA and mRNA expression, as well as protein levels, while 24 genes that were associated at least two or three of them.

**Table 2:** Significant differential expression of associated genes in cassava leaves in the iTRAQ-based proteomics database reported by previous (Zhao et al. 2015).

Marker	Cassava genome version 6.1	Cassava genome version 4.1	Arg-L1/Arg-L0	SC-L1/SC-L0	Associated Traits	Description
EME171	Manes.13G013400	cassava4.1_015024m	0.347±0.13	0.414±0.18	CAT	SMALL HEAT-SHOCK PROTEIN HSP20 FAMILY
EME254	Manes.08G095800	cassava4.1_013978m	0.944±0.97	0.221±0.05	PRO,TDMP	Ferritin
EME710	Manes.10G078800	cassava4.1_011832m	2.000±4.11	3.180±10.31	CAT, POD, TN	Caffeoyl-CoA O-methyltransferase
MeESSR46	Manes.05G090000	cassava4.1_018205m	4.067±18.94	7.510±57.53	SOD, SRS, TDMP	MLP-LIKE PROTEIN 423-RELATED

Note: Values are expressed as the average fold change in protein abundance between stressed (L1) and control (L0) leaves of Arg7 and SC124 cassava cultivars identified in the three experimental replicates. Significance thresholds were set at 1.67 and 0.6 for up and down regulation, respectively.

**Fig 5:**
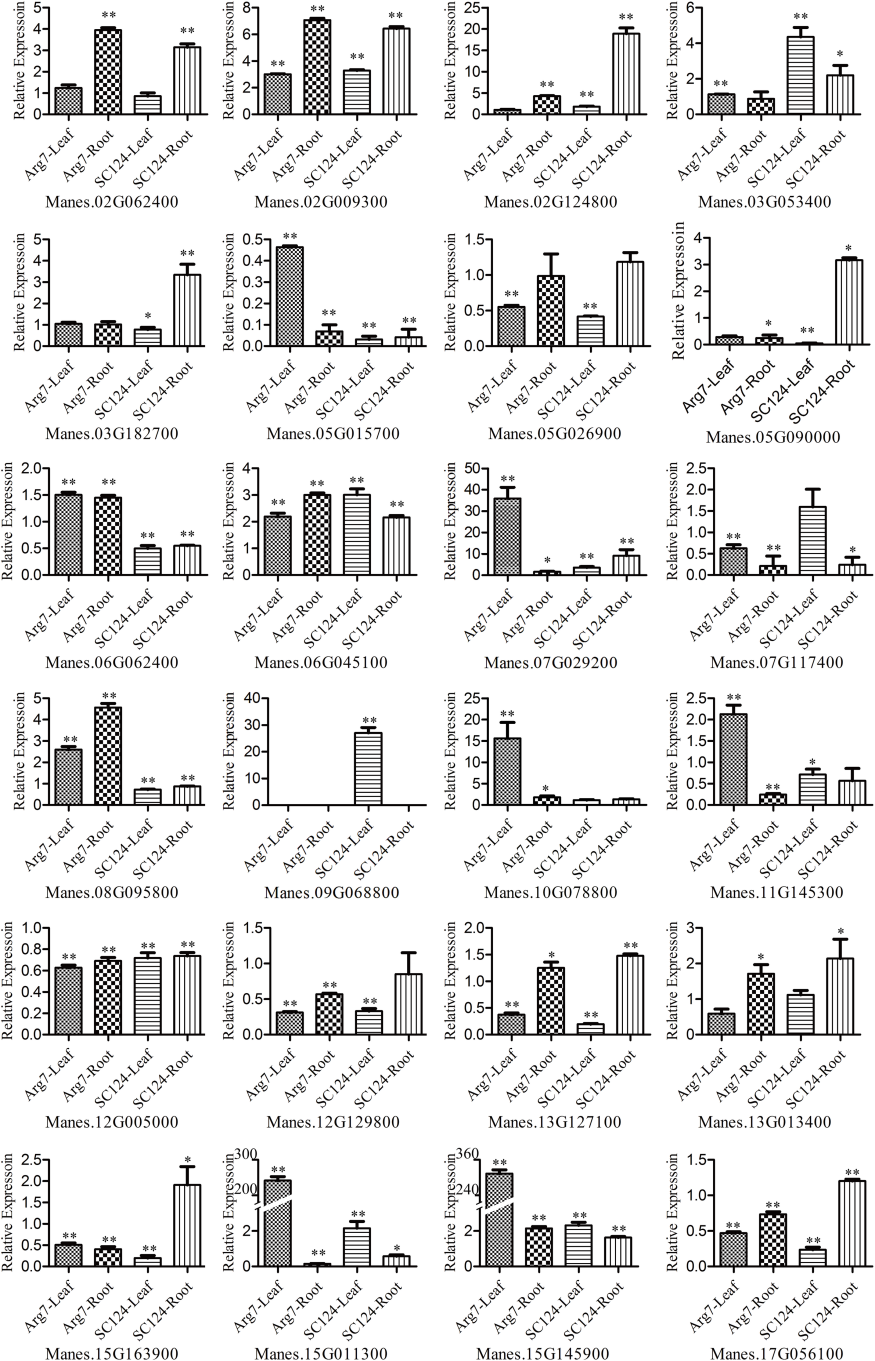
Relative expression verification of 24 selected associated genes by qRT-PCR. All of the 24 genes were selected according to the significantly differential expression from omics data. * and ** standard for significance of differential expression P<0.05 and P<0.01 respectively which were estimated from relative expression between drought and control treatments by *T* test. Manes.09G068800 was only expressed in the leaf of SC124 under drought and control conditions.

All of 24 genes were confirmed by qRT-PCR and the relative expression of every tissue compared drought to control groups were calculated and *T* test was performed to estimate whether they were significantly differential expression (*P*<0.05) or not (Fig 5). As was shown in Fig 5 and Table 3, there were 9 genes, Manes.02G009300 (co-located with MeSSR36), Manes.09G068800 (co-located with EME628), Manes.02G062400 (co-localized with EME212), Manes.02G124800 (co-localized with MeSSR38), Manes.03G053400 (co-located with MeSSR44), Manes.06G045100 (co-located with MeSSR71), Manes.07G029200 (co-located with MeSSR18), Manes.13G013400 (co-located with EME171) and Manes.10G078800 (co-located with EME710) which were significantly up-regulated expression while other 3 genes, Manes.12G005000 (co-located with EME425), Manes.12G129800 (co-located with MeSSR74) and Manes.13G127100 (co-located with MeSSR31) were significantly down-regulated expression under water stress. They were consistent with previous omics data described above and results of qRT-PCR. In addition, there were 8 genes were partly consensus with omics data while another 4 were opposite (Fig 5). All of the twenty-four differential expression genes revealed by qRT-PCR were meaningful to marker assistant selection in cassava breeding.

**Table 3:** Functional annotation of twelve differential expression genes supported by previous omics data and qRT-PCR.

Marker	Gene ID#	Tissue^a^	Associated Traits	R^2^	AAE^b^	Description	GO annotation	Enzyme	Pathway
MeESSR36	Manes.02G009300	Leaf, Root	AGFW-2015	7.90	positive	NAD DEPENDENT EPIMERASE/DEHYDRATASE	F:UDP-glucose 4-epimerase activity P:galactose metabolic process	EC:5.1.3.2	map00052,Galactose metabolism; map00520, Amino sugar and nucleotide sugar metabolism
			MDA-R-2014	3.84	negative				
EME628	Manes.09G068800	Leaf	SRN-2014	5.28	positive	AQUAPORIN PIP2-1-RELATED	C:integral component of plasma membrane P:water transport C:plasmodesma F:glycerol channel activity P:cellular water homeostasis P:glycerol transport P:ion transmembrane transport F:water channel activity C:vacuole		
EME710	Manes.10G078800	Leaf, Root	CAT-L-2014	16.75	positive	Caffeoyl-CoA O-methyltransferase	P:coumarin biosynthetic process F:caffeoyl-CoA O-methyltransferase activity F:metal ion binding P:lignin biosynthetic process P:methylation	EC:2.1.1.104	map00940, Phenylpropanoid biosynthesis; map00360, Phenylalanine metabolism; map00945, Stilbenoid, diarylheptanoid and gingerol biosynthesis; map00941, Flavonoid biosynthesis
			POD-L-2014	5.23	positive				
			SRN-2014	5.55	positive				
EME425	Manes.12G005000	Leaf, Root	AGFW-2015	16.64	positive	PROTEIN PHOSPHATASE 2C 33-RELATED	F:protein serine/threonine phosphatase activity P:protein dephosphorylation		
			SOD-R-2015	12.11	positive				
MeESSR74	Manes.12G129800	Leaf, Root	Proline-L-2015	9.13	positive	HOMEOBOX-LEUCINE ZIPPER PROTEIN ATHB-16-RELATED	C:nucleus F:transcription factor activity sequence-specific DNA binding F:transaminase activity P:regulation of transcription DNA-templated F:sequence-specific DNA binding		
MeESSR31	Manes.13G127100	Leaf, Root	Proline-L-2014	8.31	positive	CYCLIN-DEPENDENT KINASE INHIBITOR 6	P:cell cycle arrest C:nucleus F:cyclin-dependent protein serine/threonine kinase inhibitor activity P:negative regulation of protein serine/threonine kinase activity		
MeSSR38	Manes.02G124800	Leaf, Root	CAT-R-2014	3.37	negative	17.6 KDA CLASS I HEAT SHOCK PROTEIN 1-RELATED	P:response to oxidative stress, response to heat C: cytoplasm F: protein binding, protein self-association		map04141:Protein processing in endoplasmic reticulum
MeSSR44	Manes.03G053400	Leaf, Root	AGFW-2015	6.95	positive	Cation transport ATPase	P:copper ion transport, metal ion transport C: plasma membrane, chloroplast thylakoid membrane, integral component of membrane, integral component of membrane F: nucleotide binding, copper-exporting ATPase activity, copper ion transmembrane transporter activity, ATP binding, ATPase activity, coupled to transmembrane movement of ions, phosphorylative mechanism, cation-transporting ATPase activity, metal ion binding	EC:3.6.3.4	
MeESSR18	Manes.07G029200	Leaf, Root	Proline-L-2015	5.28	negative	GLUTATHIONE S-TRANSFERASE, GST, SUPERFAMILY, GST DOMAIN CONTAINING	P: gluthione metabolic process, toxin catabolic process, response to toxic substance, response to salicylic acid C: cytoplasm, cytosol F: gluathione transferase activity	EC:2.5.1.18	map00480:Glutathione metabolism
EME171	Manes.13G013400	Root	CAT-R-2015	5.34	negative	SMALL HEAT-SHOCK PROTEIN HSP20 FAMILY	P: response to heat, response to high light intensity, response to hydrogen peroxide C: chloroplast		map04141:Protein processing in endoplasmic reticulum
EME212	Manes.02G062400	Root	SOD-L-2015	15.2	positive	Domain of unknown function			
			SRFW-2014	6.21	positive				
MeESSR71	Manes.06G045100	Leaf, Root	CAT-L-2014	5.77	positive	Inorganic diphosphatase / Pyrophosphate phosphohydrolase	P:response to water deprivation, response to salt stress, auxin polar transport, establishment or maintenance of transmembrane electrochemical gradient, proton transport, leaf development C: Plant-type vacuole, mitochondrion, vacuole, vacuolar membrane, golgi apparatus, cytosol, plasma membrane, chloroplast, plant-type vacuole membrane, chloroplast envelope, endosome membrane, membrane, integral component of membrane F:inorganic diphosphatase activity, hydrogen-translocating pyrophosphatase activity, metal ion binding	EC:3.6.1.1	map00190:Oxidative phosphorylation
			CAT-L-2015	10.47	positive				
			SRDW-2014	6.91	positive				
			SRFW-2014	10.99	positive				

^a^ was the differential expressed tissue under drought stress.

^b^ was the AAEs of EST-SSR loci of associated genes for traits which they were associated to.

### Discovery of elite alleles

To estimate the phenotypic effects of alleles and their AAEs, we used 24 loci co-locating with genes were significantly differential expression under water stress confirmed by qRT-PCR as target for elite allele’s discovery, as previously reported [38]. As were shown in S5 Table, all of these 24 associated loci contained 33 marker-trait combinations and 87 allele-trait combinations, and these 87 allele-trait combinations also included 34 positive and 53 negative alleles. All of these positive alleles except alleles which were associated with MDA content were elite alleles while negative alleles associated with MDA content were elite alleles because of MDA effects to drought tolerance. We obtained the typical materials that carried elite alleles were obtained (S5 Table). Finally, total of 23 positive and 10 negative AAEs were identified.

## Discussion

Drought tolerance is a complex trait and difficult to study for it’s more affected. We performed the water stress experiments by using 100-day old plants of cassava germplasms for their suitable size of plants, being convenient to control the water stress condition in the green house and 100-day old also is a key time point for cassava storage root development and enlargement. So, we could not only evaluate the survival rate but also potential of biomass and storage root development and enlargement of cassava under water stress. Besides, it is valid for multiple crops that water stress experiment is performed in their seedling stage [39–45].

Molecular diversity, water depressed experiment and association mapping in our study were based on 134 cassava genotypes. Although 134 genotypes are too little for a large scale GWAS study, it was difficult to water stress experiment of cassava, a big plant crops, in greenhouse and our researches should be considered as a candidate association mapping strategy. Besides, results of association mapping in our study were valid which has been verified by drought-related transcriptomics and proteomics of cassava and qRT-PCR and many other direct or indirect evidences.

### Genetic diversity and population structure

All 107 EST-SSR markers used in this study were localized in all 18 cassava chromosomes that had potential to analysis of genetic diversity and population structure. Compared with previous studies on genetic diversity of cassava germplasms, the number of alleles per locus in our study was lower than that previously reported for 42 cassava land races in Brazil [46]. This might be due to the EST-SSR markers used in our study, that is, 55 previously reported EST-SSRs [5] and 52 EST-SSRs which were developed against unique polymorphism [8], were naturally lower abundant polymorphisms SSRs than genomic SSRs. Genetic coefficients between every two accessions were very low on average with a wide range. These indicated that genetic relationships between these germplasms were very complex and should due to the intricacy of their original areas and breeding history. However, AMOVA of germplasms showed that sub-populations, dependent on geographical origin, were not significantly different and had a strong gene flow among them. That might a consequence of the cassava introduction in breeding projects.

Results of population structure analysis in our study showed that all 134 germplasms could be divided into three clusters which we evaluated by AMOVA, finding significant differences and a strong gene flow. However, there were not obviously relationship between clusters and origin area. While the germplasms themselves derived from different areas of the world, most of them came from China, while east-south Asia and Africa, two of major cassava producing regions, and were rare in these accessions. Moreover, cassava land races from China represents mostly outbred offspring derived from parent plants from other countries.

The population differential coefficients were lower than those of the 283 cassava samples calculated by Fregene et al. [47] and that of 36 African cassava land races reported by Raji et al. [48]. This indicates that the 134 cassava germplasms used in our study are suitable for association mapping based on linkage disequilibrium because of that their population differentiation was not very strong as well as abundant genetic and phenotypic diversity.

### Marker-trait association mapping

In our study, fifty-three EST-SSRs located on 17 chromosomes and accounted for 54.08% of 98 markers. In order to understand the EST-SSRs used in our study, we obtained the reference EST sequences and genebank EST library (S6 Table). Nine EST libraries contained all 107 EST-SSRs and three of them sequenced by previous reports aiming to identify the different water or abiotic stress tolerance related genes in cassava cultivars [49–51]. 96 EST-SSRs derived from these three libraries, accounting for 89.7% of 107 markers. This allowed us to map functional genes for drought tolerance by association mapping and this might be the reason why so many loci were associated with drought-related traits in our study.

Just as reported in other crops before, we found markers for repeat, pleiotropy markers and one-to-one phenotype-marker association combinations in this study [23, 52–53]. Twenty-two major marker-trait combinations (R^2^>10%) were explored, accounting for 25.29% of all combinations. Seventeen markers associated with multiple phenotypes in one year. Moreover, we found thirteen pleiotropic loci in two years that were not only associated with biomass and storage root related traits of 100-day old plants, but also with drought tolerance. All of these genes are potential candidate genes for drought tolerance and high yield breeding in cassava.

Due to few related reports in cassava, it was difficult for us to compare our results with previous researches. Instead, we compared our results to cassava drought related transcriptomics and proteinomics and performed qRT-PCR to verify differential expression genes. Markers associated mean that genes they co-localized with the map or their neighboring chromosome regions (within covering ± LD decay distance) regulated the development of phenotypes they were associated to, meanwhile alleles of actual functional genes had different phenotypic effects. However, it was not to say any EST-SSR and enzymes/molecules co-localized with the map location of identified candidate genes. Expression analysis and functional annotation were two effective methods of the real functional candidate gene discovering for associated region.

Some associated genes or their orthologues or paralogues have similarly functions in cassava or other model plants. MeSSR50 associated with SRS content in leaf in 2014 in our study. According to previous reports [20, 54], 14-3-3 protein family might play roles in cassava storage root enlargement stage and be related to the starch and sugar metabolic and drought responsive. That indicated that our results were supported by previous reports. Another EST-SSR marker, MeSSR18, associated with proline content. The marker which located in the gene annotated as *MeGSTU7*, with gene code ID Manes.07G029200, whose expression rose after drought treatment for 12 days. The expression of one of its paralogous genes improved after by drought stress and recovered to normal after cassava plants were re-watered. A previous report linked *MeGSTU7* to AGFW [55]. Although results of our study and previous reports [55] were not completely consistent, the expression pattern of the two member of the *GSTU7* gene family in cassava were similarly under drought condition. Furthermore, *Arabidopsis* orthologues of associated genes discovered in our study played roles in water stress or abiotic stress resistance, such as H(+)-translocating (pyrophosphate-energized) inorganic pyrophosphatase (AtVHP1)[56], heat shock protein (AtHSP21)[57], homobox protein 16 (HB16)[58], decapping 5 (DCP5)[59]. These supported loci had potential to be considered as candidate genes for further functional verification and tag markers in MAS project for cassava drought tolerance and yield breeding.

### Discovery of elite alleles

In order to explore the elite alleles in associated loci, we estimated AEEs of 24 associated loci whose expression changed under drought condition, indicating that they might be functional genes (S5 Table). POD, SOD, CAT, Proline and SRS were positive factors and MDA as negative on the basis of their contribution to drought resistance. We defined elite alleles as alleles positively influencing drought tolerance and high yield breeding, therefore loci associated with POD, SOD, CAT, Proline, AGFW and storage root-related traits and a negative association with MDA. We found three elite alleles as for MDA content and two associated loci were negative effect according to their AAE values. All phenotypes and AAEs of these could be useful for molecular assisted selection and pyramiding breeding in the future.

These alleles, whose phenotypes comprising multiple traits were consistent with comprehensive breeding targets for drought tolerance and high yield breeding in pleiotropic loci, might be valuable for future research. For example, EME710 associated with SRN, CAT and POD activity, with positive AAEs for three traits. Moreover, the allele B in EME710 was an elite allele according to our definition. There were another two loci like EME710 of all 9 casual pleiotropic loci. Another 4 loci did not show elite allele characteristics for all associated phenotypes. Among them, MeSSR36 was associated AGFW and MDA content. As mentioned above, MDA content is negative factor for drought tolerance. Therefore, allele B in MeSSR36 was the allele to consider for drought tolerance and high yield breeding because of its positive effect on AGFW content and negative on MDA content. All of these pleiotropic variations have a potential application for comprehensive breeding.

### Cross-talk about associated mapping and expression analysis

Compared to associated genes and omics data and results of qRT-PCR by using independent drought stress experiment, 24 differential expression genes were obtained under water stress. Twelve of them selected for their consensus in multiple independent experiments would be actual functional genes of cassava drought resistance. Furthermore, amino acid sequences of them were used to functional annotation. And we tried to explain part of cassava drought resistance mechanisms revealed by these functional genes.

As was shown in Table 3, Manes.02G009300 (co-localized with MeSSR36) was annotated to UGE5 protein (EC: 5.1.3.2) and might regulate the galactose metabolism (map00052) and amino sugar and nucleotide sugar metabolism (map00520). Homolog genes of it in *Arabidopsis thaliana* were reported to regulate cell wall biosynthesis. *AtUGE2* and *AtUGE4* were synergetic to regulate galactose content of cell wall which was correlated to shoot growth. *AtUGE5* was slightly cooperated with *AtUGE4* to regulate the root growth and galactose content which might be induced by stress [60]. In our study, *MeUGE5* was associated with DTCs of AGFW and MDA and was significantly up-regulated expression. So, we indicated that *MeUGE5* might promote shoot growth and enhance the cell membrane stability. Besides, *MeUGE5* was a positive effect locus for DTC of AGFW but negative one for DTC of MDA content according to AAE. These might support our speculation.

Manes.10G07880 (co-localized with EME710) which was annotated to Caffeoyl-CoA O-methyltransferase (EC: 2.1.1.104) might regulate multiple metabolism pathways and flavonoid biosynthesis pathway was one of them. Flavonoid biosynthesis pathway was considered as one of important active oxygen elimination mechanisms under stress condition [61]. In our study, it was associated with DTCs of CAT, POD and SRN with positive effects and up-regulated under drought stress. All of the above indicated that Manes.10G078800 might participate in the active oxygen elimination during cassava drought resistance process.

The third up-regulated gene Manes.09G068800 (co-localized with EME628) was annotated to AQUAPORIN PIP2-1-RELATED which was intrinsic protein of cell membrane and had glycerol channel activity and water channel activity. Its homologous gene in *Arabidopsis* played role in water absorption of root. And in our study, its expression patterns under water stress agreed with its possible functions. It was indicated that up-regulation of *MePIP2-1* might enhance the water up-take ability of cassava root and have positive effects on DTC of SRN and cassava drought resistance. But it was still unknown for us how *MePIP2-1* regulates DTCs of SRN of cassava under drought stress.

Besides the three genes, another two genes, Manes.03G053400 and Manes.06G045100 which were annotated as positive regulators [62, 63] were up-regulated under drought stress with positive AAEs (Table 3). However, there were 3 genes, Manes.02G124800. Manes.07G029200 and Manes.13G013400, which were annotated as positive regulators [55, 64–65] for drought resistance were up-regulated under water deprived condition with negative AAEs for their associated traits (Table 3). That was different from our knowledge and might due to functional alleles and complex relationships of their associated traits and pathways these genes directly participated in.

There were three down-regulated expression genes under water stress, Manes.12G005000 (co-localized with EME425), Manes.12G129800 (co-localized with MeSSR74) and Manes.13G127100 (co-localized with MeSSR31), that were annotated to *MePP2C33*, *MeHB16* and *MeKRP6* respectively. PP2C proteins were negative regulators of ABA pathway and stress-induced MAPK signaling pathway and they also involved in regulation the cell cycle [66, 67]. *MeHB16* was a member of the HD-ZIP super-family and its homologous gene *AtHB16* acted as a negative regulator of cell expansion, leaf growth and photoperiod of *Arabidopsis* [68]. As was shown in Fig 5, *MePP2C33* and *MeHB16* were down-regulated in Arg7 root under drought stress while not significant changed in SC124. Besides, *MePP2C33* was associated with DTC of AGFW with positive effect of AAE. That might account for the continuous growth of Arg7 under drought condition reported previously [20]. As results of GO annotations, *MeKRP6* might participate in cell cycle arrest and negative regulation of protein serine/threonine kinase activity process. The energy sensor *AtSnRK1* plays a cardinal role in the control of cell proliferation in *A*. *thaliana* plants through the inhibition of *AtKRP6* biological function by phosphorylation [69]. However, *MeKRP6* was down-regulated in leaf tissue of SC124 under drought stress in our results which could be explained for its function and expression pattern but not for the difference between cassava cultivars. All of these three negative regulators were down-regulated under water depressing condition which indicated that these genes might regulate the continuous growing ability of cassava plant under drought stress. Differential expression patterns between *MeKRP6* and the other two genes in cassava cultivars might be related to the different strategies of cultivars for drought resistance. As was shown in Table 3, AAEs of these negative regulators were positive. That might be for their expression patterns under drought stress or loss of function in natural alleles of these negative genes. However, it was difficult to explain the mechanisms based on evidences at present that these down-regulated genes were associated with phenotypes under drought stress but it was no doubt that they were actual functional genes under drought stress.

## Conclusion

In conclusion, all of these 134 cassava accessions showed abundant genetic and phenotypic diversity and a slight sub-population differentiation. The results of association mapping were supported by reverse genetics evidences as well as previous study, allowing selecting elite alleles of the associated loci. These results will make possible to choose cassava resources for drought tolerance and to provide plant materials, candidate genes and reliable experimental support for further research in the mechanism behind cassava drought tolerance.

## Supporting information

S1 Fig Mapping of the 98 EST-SSRs used in this study on cassava chromosomes. (TIF)

S1 Table The pedigree of all the 134 cassava accessions. (XLSX)

S2 Table Information of primers used in qRT-PCR. (XLSX)

S3 Table Summary statistics of drought tolerance coefficient of phenotypes in water stress expreiments. (XLSX)

S4 Table Results of marker-trait association mapping in cassava germplasm resources (P_FDR<0.05). (XLSX)

S5 Table Phenotypic effect and their typical materials of alleles and AAE of locus in 24 reliable associated loci. (XLSX)

S6 Table Reference EST sequences and genebank EST libraries 106 cassava EST-SSRs derived except one marker. (XLSX)
