# Supplementary figures and images for: Molecular diversity analysis, drought related marker-traits association mapping and discovery of excellent alleles for 100-day old plants by EST-SSRs in cassava germplasms (Manihot esculenta Cranz)
Source: PLoS One. 2017 May 11;12(5):e0177456. doi: 10.1371/journal.pone.0177456 (PMC5426748; doi:10.1371/journal.pone.0177456)

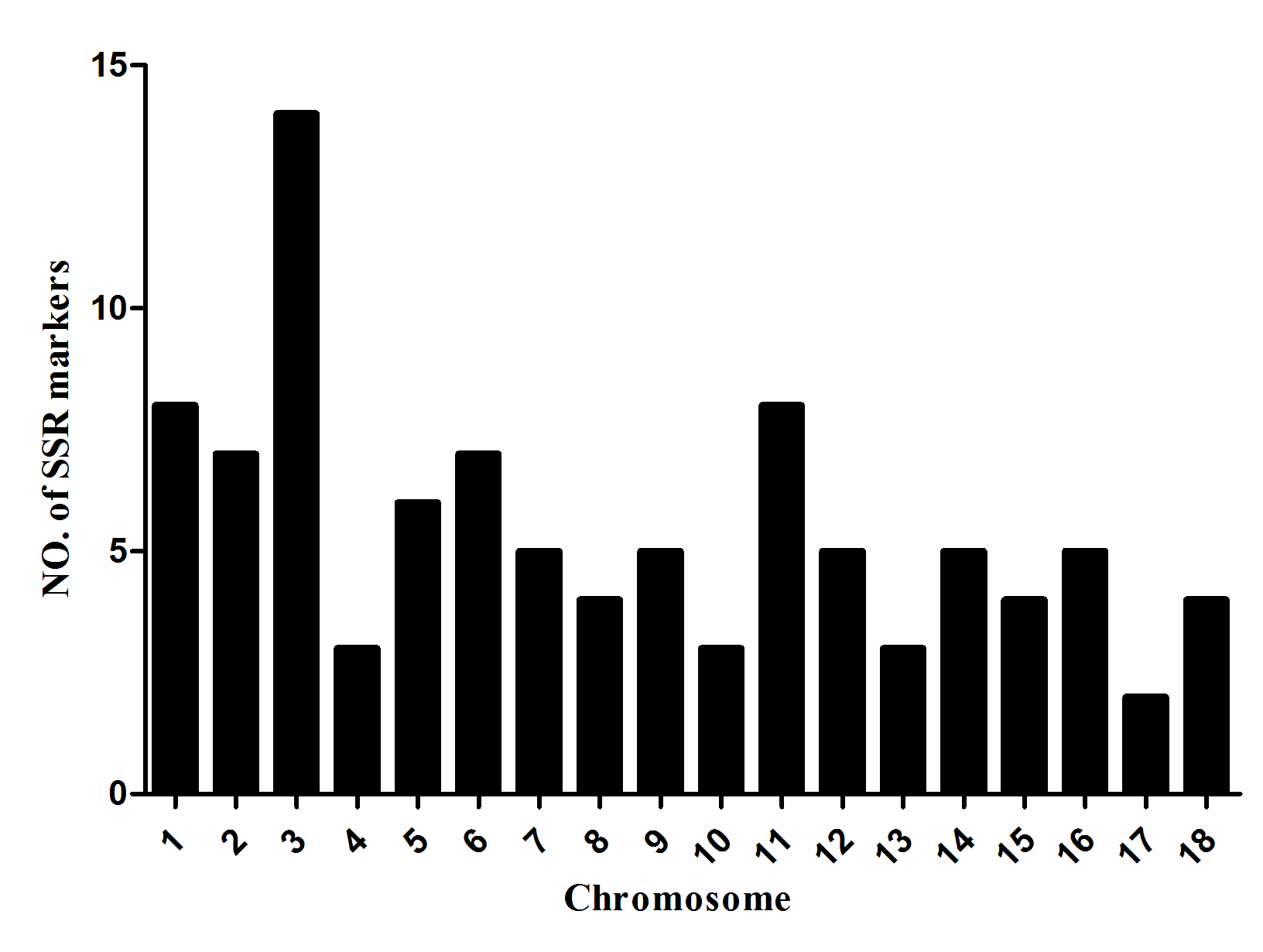

Supplement: S1 Fig — (TIF) [file pone.0177456.s001.tif]
